# RUM/FILH: a standardized operational capability model for biodiversity databases

**DOI:** 10.1093/database/baag044

**Published:** 2026-07-28

**Authors:** Miklós Bán

**Affiliations:** HUN-REN DE Behavioural Ecology Research Group, Egyetem tér 1, 4032, Debrecen, Hungary; Department of Evolutionary Zoology, University of Debrecen, Egyetem tér 1, 4032, Debrecen, Hungary

## Abstract

The increasing reliance of biodiversity research on large-scale databases has brought significant progress in data accessibility but also new challenges in data comparability, reliability, and interpretability. While global platforms, such as GBIF and iNaturalist standardize and disseminate vast quantities of biodiversity information, they cannot fully meet the needs of users relying on local or thematic data sources. In this paper, we propose a unified service capability model, the RUM/FILH model, that provides a transparent, comparable framework for describing database functionalities and data management quality. The RUM model distinguishes three essential data-related services: Read, Upload, and Modify, while the FILH extension integrates FAIR principles and data history accessibility. This combined system enables both users and database administrators to assess interoperability, metadata quality, and licensing conditions quickly and consistently. We demonstrate the applicability of the RUM/FILH model across biodiversity databases of different scales, including international aggregators and community-based platforms. The proposed capability model enhances trust, transparency, and collaboration across distributed data infrastructures, supporting the development of a more coherent and sustainable global biodiversity informatics network.

## Introduction

In recent decades, biodiversity and ecological research has increasingly become a data-driven science [1–3], which has required the technical and collaborative development of databases. As a result of this process, the organization of data into databases has led to the emergence of increasingly large, cross-border systems and the development of data integration or aggregator databases. Large international databases, such as GBIF and iNaturalist play a key role in this transformation process [4,5], albeit in different ways, as they make huge amounts of data available to researchers in a uniform format and provide data management and collection tools that can be used by anyone. The standardization of data facilitates widespread reuse [6], which can lead to previously unimaginable large-scale, even global-scale analyses (e.g. [7–10]). At the same time, the services of large databases do not cover all user needs and have limitations that affect the interpretation of data and may influence the quality of research results (e.g. [11–14]). In the case of larger, global data collections, especially in the case of community science-based systems, data quality [15,16] poses a significant challenge, while in the case of large aggregator databases, most of the challenges stem from spatial and taxonomic biases, as well as the heterogeneity of sources [17–19]. These problems affect the reliability of models derived from the data and the interpretability of spatial patterns of biodiversity [20,21]. Smaller, often project-based databases are usually criticized for their uncertainty in terms of their lifespan and long-term sustainability [22].

Criticisms and suggestions regarding the size and operating model of databases can be found in the literature [22–25], and it is increasingly recognized that the idea that a distributed network of databases would best serve diverse usage needs, with appropriate regulation, is becoming accepted [26–28].

In order to facilitate the establishment of links between databases and to help users navigate the increasingly complex and growing volume of data and services available, we need simple solutions that are easy to understand and use in everyday practice. Various labels, such as quality indicators, are a big help here [29–31]. However, current labelling systems focus primarily on data, while labels related to the processing of information about the operation of data management systems by users or automated processing have not become widespread.

The development and use of such a label representation of the operational capabilities are motivated by everyday practice in biodiversity informatics, where users interact not only with large aggregator databases but also with their source databases and with databases that are only partially—or not at all—connected to the global biodiversity data infrastructure. These source databases are often local or thematic systems designed to meet the needs of specific communities and projects. As a result, they differ considerably in their services, accessibility, interoperability, and data management practices, making direct comparison difficult.

Some initiatives already provide mechanisms for evaluating the quality and trustworthiness of data repositories. For example, CoreTrustSeal offers certification for repositories that meet established requirements for long-term preservation and reliable stewardship of research data. Similarly, FAIR-assessment initiatives and services such as FAIRassist support repository managers and software developers in implementing and evaluating FAIR principles.

However, these initiatives primarily focus on repository quality, trustworthiness, or FAIR compliance. They do not provide a simple and standardized description of how databases operate from the perspective of everyday users and potential partner databases. In particular, they do not offer a concise way to communicate practical characteristics, such as data accessibility, upload permissions, modification rights, interoperability options, licensing conditions, or the availability of data history.

To address this gap, we propose a standardized service-capability-labelling model for biodiversity databases. Rather than serving as a rating or certification system, the proposed framework provides a concise description of database functionalities and services. Such standardized labels can facilitate comparisons among biodiversity databases of different sizes and purposes, support the establishment of links between databases, and help users quickly understand the capabilities and limitations of the services provided.

## The importance of professional databases of primary biodiversity data and their relationship with aggregator databases

Nonaggregating, i.e. primary data collection databases (data collectors are mostly data providers), whether local, regional, or national data management systems, as thematic (and generally small) databases, are of paramount importance in that they provide data collection and management interfaces for local data collection programs, which would not be able to function without these interfaces [32–34]. Personal relationships are of paramount importance in these projects, both in terms of data accessibility and data acceptance or modification [35,36]. Since the data processed is primary and there is a direct relationship between data collectors and curators, the chance of incorrect data use due to misinterpretation is low in local-level data use, and data quality assurance can also be reliably implemented through personal relationships. Although such local and thematic databases are important collection points for local biodiversity observation data, it is also crucial that these databases establish links with larger aggregator systems, thus ensuring the long-term preservation and findability of data [24,26] on a wider scale.

In source databases, if they provide public access to their data, it may be important for users to know whether all records and all their attributes are available to them. When establishing data connections, it is important to consider whether all information is transferred from the source database to an aggregator database, or whether there are attributes that remain available only in the source database.

After establishing data connections and publishing data sets, the management of data extracted from primary data collection databases changes accordingly, as the data typically does not change in the aggregator database. Modifying data and tracking the resulting changes is a fundamental and critical operational feature of a primary biodiversity data collection database. Data modifications are often necessary and are performed either by curators or data collectors, or in some cases, users may modify each other’s data records. The development of data modification options is fundamentally a matter of trust in a database, which is likely to be more limited the more receptive the database is to incoming data. If data changes in the source database, it may not be transferred back to the aggregator database, which in practice creates noticeable differences between source databases and aggregators [37] and can make it significantly more difficult to track the history of data.

After the data has been transferred, it may become available to users in several independent locations (source database, aggregator databases) with slightly different content (e.g. as a result of standardization), structure, and appearance, and even with different access rules. Although it is in the interest of both sides (source and aggregator) to minimize these differences, there are also factors such as information on endangered species or personal data that are deliberately removed or modified during the transfer process, which raises the question of how to most clearly inform users about possible differences.

## Recommendations for promoting the establishment and maintenance of cooperation between biodiversity databases with different purposes and levels

Biodiversity data infrastructures increasingly consist of interconnected but structurally heterogeneous systems, ranging from small, project-based or thematic databases to large international aggregators. Although these systems are often linked through data exchange pipelines, their operational behaviour differs substantially, particularly with respect to data accessibility, modification rights, versioning, and interoperability constraints. These differences are not always transparent to users or to partner systems, even when technical documentation is available.

A key structural limitation in current biodiversity data integration practice is that interoperability is typically defined at the level of *data exchange formats and standards* (e.g. Darwin Core, API specifications), while the *operational semantics of data services* (that is, how data can be read, uploaded, modified, and traced across systems) remain implicit. As a result, users and institutions making integration decisions often lack a comparable and concise description of how a given database behaves as a data service, rather than what data it contains.

This lack of operational transparency becomes particularly critical in distributed infrastructures where data are not static. Once data are transferred from primary databases into aggregators, updates, corrections, and metadata refinements in the source system may not propagate consistently, or may propagate only partially. Consequently, identical datasets may exist in multiple locations with differences in structure, completeness, update status, and licensing interpretation. These divergences are often not explicitly visible to end users, even though they directly affect reproducibility, traceability, and analytical reliability.

Addressing this issue requires not only improved data standards, but also a lightweight mechanism to describe and compare the service-level behaviour of biodiversity databases. Such a mechanism should be independent of repository size, governance model, or institutional role, and should remain simple enough to be applied consistently across heterogeneous infrastructures.

We therefore argue that strengthening cooperation between biodiversity databases of different scales and purposes requires an additional descriptive layer that complements existing metadata and FAIR-related assessments. In addition to financial, technical, and capacity-building support for smaller databases, interoperability can be significantly improved by introducing a standardized service-labelling system that describes core operational properties of databases in a comparable format.

## The RUM (FILH) model as a best practice for comparing databases and monitoring data status

Databases, ranging from the narrowest thematic databases to regional and national collection databases and the largest international aggregators, provide their users with data-related services under varying conditions, which relate to the uploading, reading, and modification of data. These options, defined by rules, are not easily transparent to users, even if they are well documented. However, these are parameters that can be determined empirically for any database, making these services comparable regardless of the database’s operating model and size. For this comparison, we recommend using the RUM model (a description of basic interaction capabilities), which distinguishes three fundamental capabilities: reading (R), uploading (U), and mutation (M). All three capabilities are characterized by the extent to which external actors can access them. This extent is indicated by three colours, in the following order: no access (red), restricted access (black), and open access (green). These are not permissions, they are system affordances. R (Read capability): ability of external agents to access data under defined conditions (none > restricted > open). U (Extend capability): ability of external agents/systems to contribute data into the system. M (Mutation capability): ability to alter existing records, including who can modify and under what governance rules.

This descriptive system allows for a transparent comparison of the data lifecycle policies of different databases using a simple coloured string notation that makes it easy to label databases. Examples of labelling and explanations of labels in Table 1:

**Table:** 

• iNaturalist:	R (black)	U (green)	M (black)
• GBIF:	R (black)	U (black)	M (black)
• eBMS:	R (red)	U (black)	M (black)
• PLANTS Database:	R (green)	U (red)	M (red)
• Mushroom Observer:	R (green)	U (green)	M (black)

**Table 1:** Justification for RUM labelling of examples.

Label	Explanation of labelling: iNaturalist (https://inaturalist.org)
R (black)	The coordinates of observations may be hidden or obscured (https://help.inaturalist.org/en/support/solutions/articles/151000169938-what-is-geoprivacy-what-does-it-mean-for-an-observation-to-be-obscured-). You must log in to export records (https://help.inaturalist.org/en/support/solutions/articles/151000170342-how-can-i-download-data-from-inaturalist-), but most of the data and additional attributes of the records are publicly available, as anyone with an email address can register for free (https://help.inaturalist.org/en/support/solutions/articles/151000195690-how-to-sign-up-for-an-inaturalist-account).
U (green)	Only logged-in users can upload data, but anyone can register for a user account (https://help.inaturalist.org/en/support/solutions/articles/151000192921-how-to-make-an-observation, https://help.inaturalist.org/en/support/solutions/articles/151000195690-how-to-sign-up-for-an-inaturalist-account).
M (black)	Data providers can freely modify their own data, but cannot modify the data of others (https://help.inaturalist.org/en/support/solutions/articles/151000197239-inaturalist-iphone-app-the-edit-observation-page, https://help.inaturalist.org/en/support/solutions/articles/151000175695-what-are-licenses-how-can-i-update-the-licenses-on-my-content-,).
Label	Explanation of labelling: GBIF (https://gbif.org)
R (black)	Access to data classified as sensitive may be restricted in several ways (https://docs.gbif.org/sensitive-species-best-practices/master/en/).
U (black)	Only institutions registered as publishers or GBIF partners can upload data (https://www.gbif.org/become-a-publisher, https://ipt.gbif.org/manual/en/ipt/latest/how-to-publish).
M (black)	Only data providers can modify data via the IPT (https://ipt.gbif.org/manual/en/ipt/latest/manage-resources).
Label	Explanation of labeling: eBMS **(**https://butterfly-monitoring.net/**)**
R (red)	The data can be freely browsed on a map, but cannot be downloaded or accessed in detail. Only the species name and date are visible attributes in the observations. The location of the observation is shown on the map. There are no records that are completely freely accessible in their entirety (https://butterfly-monitoring.net/elastic/all-records, https://butterfly-monitoring.net/index.php/ebms-data%20access, https://butterfly-monitoring.net/sites/default/files/eBMS%20DATA%20REQUEST%20POLICY%20%3D%20Annex%20B%20v2019%2004%2001.pdf). It should be noted that some participating countries also publish their data on GBIF (e.g. https://www.gbif.org/dataset/caa38f94-ddeb-43e7-9a27-1ac46d7ddc5c), but this does not change the red R mark, because the data cannot be downloaded from the eBMS database interface. Registered users can download their own data.
U (black)	Only registered users can upload data. Anyone can register, but there are additional conditions for who can be a data provider (https://butterfly-monitoring.net/user/register, https://butterfly-monitoring.net/index.php/mydata).
M (black)	Data may be modified by data collectors and possibly coordinators (https://butterfly-monitoring.net/sites/default/files/eBMS%20DATA%20REQUEST%20POLICY%20%3D%20Annex%20B%20v2019%2004%2001.pdf).
Label	Explanation of labeling: PLANTS Database **(**https://plants.usda.gov/**)**
R (green)	Raw data is not provided, but the checklist and species distribution data can be downloaded without restriction (https://plants.usda.gov/downloads, e.g. https://plants.usda.gov/plant-profile/AIAL).
U (red)	Data upload options are not available.
M (red)	Data modification options are not available.
Label	Explanation of labeling: Mushroom Observer **(**https://mushroomobserver.org**)**
R (green)	The data stored in the database is accessible to anyone after logging in. Anyone can register in the database without restriction (https://mushroomobserver.org/articles/46).
U (green)	Registered users can upload new data without restriction (https://mushroomobserver.org/info/how_to_use).
M (black)	Registered users can add comments to uploaded data without restriction and supplement them, but they cannot modify existing attribute values (https://mushroomobserver.org/info/how_to_use).

Building on the RUM framework, we introduce the FILH extension (extended interoperability capabilities) integrating FAIR and data history principles. Nevertheless FILH does not redefine FAIR; it maps selected FAIR dimensions into operational observables. Similar to the previous model, FILH can be interpreted using three colors to indicate the level of its implementation. The F (Findability capability) indicates the presence or absence of metadata. It can only be marked green if the metadata of the data complies with one of the metadata standards (currently mostly Darwin Core). The (F) mark is given a black colour if the metadata is optional or of uneven quality, which hinders the retrieval of data. Red (F) indicates the absence of metadata, through which the data could be found using metadata search engines. The F.A.I.R Accessibility parameter is described by ‘R + U’ in the RUM model, so no additional marking is required. The F.A.I.R Interoperability marking has the same marking (I) in the FIL model and serves to accurately indicate the capabilities of interoperability in automations (Green: two-way data connections can be established, Black: one-way, i.e. partial data connections can be established, Red: no API and no documented data connection options). Finally, we recommend the L (Licensing/reuse) capability indicator to indicate the realized applicability of F.A.I.R Reusability, the colours of which depend on the existence and type of license indicating clear usability. Green (L) indicates that there is a license and that it is clearly permissive, requiring users to cite the source of the data at most. Any restrictive license is marked with black (L). The absence of a license or a license that prohibits use is marked with red (L). Finally, we also recommend using a label for the data history H **(**History/traceability capability), indicating whether the ability to reconstruct state transitions of data objects over time. Data with a version history that is accessible to users and suitable for automatic processing can be marked with a green (H) letter. If the data has a version history but it is limited, unclear, incomplete, or cannot be processed automatically, it is marked with a black (H) letter. Finally, if the data does not have an available version history, it is marked with a red (H) letter.

Example of FILH model application in the case of Mushroom Observer:

F (red): No metadata.

I (black): There is a bidirectional API, but it is not documented.

L (green): All observations and attached images are published in the database under some form of CC license (https://mushroomobserver.org/info/how_to_use).

H (green): The data has a version history and can be automatically processed using the active-logs RSS feed (https://mushroomobserver.org/activity_logs/rss).

Finally, the RUM/FILH marking can also be provided in a machine-readable format by replacing the color codes with +−0 signs, where − represents red, 0 represents black, and + represents green. For automatic machine recognition, we recommend using a clear pattern in square brackets in a 3 + 4 character format, separated by a right-leaning slash. Using the example of Mushroom Observer, the complete RUM/FILH marking suitable for machine reading looks like this: [++0/−0++].

The RUM/FILH model is easy to apply, allowing anyone to determine relatively easily which labels should be applied to a given database.

However, if a user performs the tagging, they may arrive at a different result than if database administrators performed it, because understanding the operations behind each tag depends significantly on the amount, transparency, and quality of the documentation.

For this reason, the most acceptable solution would be to have a public shared space for tags, where anyone can modify and justify the tags. Thematic database lists can be found in several places on the internet, among which the lists created and maintained by the Wikipedia community are particularly up-to-date. In this way, the most practical solution seems to be the creation of a RUM/FILH Wikipedia page that can link database lists and databases’ own wiki pages and access URLs, as well as ensure the public creation and maintenance of tags.

## Conclusion

Large international online databases are of undeniable value in research, but their use may be limited and they are not able to satisfy all user needs. Local, thematic biodiversity databases play an important role in data collection and in the development and maintenance of data-collecting communities, as they are more flexible, more personal, and in some cases provide a more reliable source of data than large aggregator databases. Local primary biodiversity databases are often developed to serve the specific needs of particular communities, projects, or regions. As a result, they differ substantially in their governance, workflows, and technical implementation. Complete standardization across such systems is neither realistic nor desirable. Instead, effective interoperability requires a common framework that can describe heterogeneous databases in a consistent and comparable manner without constraining their internal operation. The operational capability model we propose can help both developers who want to build connections between databases and researchers who collect data from multiple databases. For users, these label represeantations can help to get a preliminary overview of the possibilities offered by the services provided by the database. Without a standardized description of database operational behaviour, interoperability decisions are made on incomplete, inconsistent, and noncomparable information across biodiversity infrastructures.

Strengthening cooperation between aggregators and primary data collection systems of various sizes, as well as promoting a culture of data management, can significantly contribute to the flow and use of biodiversity data. Existing frameworks (FAIR, CoreTrustSeal, etc.) describe either the properties of the data or the compliance of repositories, but do not provide a unified representation of the operational capabilities of data services. RUM/FILH addresses this gap by defining a minimal capability model that is implementation-independent and focuses on interaction semantics.

The RUM/FILH model can provide practical support for the more flexible and efficient management of biodiversity data. In doing so, it can support the the transition from isolated biodiversity databases towards a more transparent and interoperable network of interacting data services.

Although the RUM/FILH model is easy to apply, the lack of a certified management and control process can lead to inconsistent or inappropriate labels. To avoid this, we recommend attaching the label creation workflow justification to the labels for each database, which can serve as a guarantee for label harmonization between institutions.

## Funding

None declared.
